# Pericardial Sarcoma of Uncertain Origin in a Young Cat: A Case Report Highlighting Diagnostic Challenges and Thoracic Drainage in Palliative Management

**DOI:** 10.3390/ani15233486

**Published:** 2025-12-03

**Authors:** Miki Hirose, Kazumi Shimada, Aki Takeuchi, Kazuyuki Terai, Aimi Yokoi, Ahmed Farag, Akari Hatanaka, Rio Hayashi, Marino Hosoki, Daigo Azakami, Rina Nabeta, Ikki Mitsui, Lina Hamabe, Ryou Tanaka

**Affiliations:** 1 Veterinary Teaching Hospital, Tokyo University of Agriculture and Technology, Fuchu 183-8509, Tokyo, Japan; mikihirose96@gmail.com (M.H.); akkiki89@gmail.com (A.T.); terai.kazuyuki.dvm@gmail.com (K.T.); aimi.y139@gmail.com (A.Y.); ahmedfarag9331@gmail.com (A.F.); akarihtnk12@gmail.com (A.H.); s196832r@st.go.tuat.ac.jp (R.H.); hosoki.m07@gmail.com (M.H.); 2 Veterinary Clinical Oncology, Tokyo University of Agriculture and Technology, Fuchu 183-8509, Tokyo, Japan; azakami@go.tuat.ac.jp; 3 Royal Veterinary College, University of London, London NW1 0TU, UK; rnabeta@rvc.ac.uk; 4 No Boundaries Animal Pathology, LLC, Fuchu 183-0053, Tokyo, Japan; mitsui@no-boundaries.jp

**Keywords:** cat, echocardiography, cardiac tumor, undifferentiated pleomorphic sarcoma, computed tomography, pericardial effusion, pericardial sarcoma

## Abstract

Primary cardiac tumors are uncommon in cats, with pericardial sarcomas being exceptionally rare. A 1-year-old spayed female cat presented with dyspnea secondary to severe pericardial effusion. Imaging revealed a pericardial mass, initially suspected as malignant mesothelioma. Cytology of the effusion was nondiagnostic, prompting partial pericardiectomy and biopsy. Histopathology and immunohistochemistry (vimentin-positive, cytokeratin-negative, Iba-1-negative) confirmed an undifferentiated sarcoma. Necropsy demonstrated diffuse tumor infiltration into the pericardium, myocardium, and pulmonary vasculature. Thoracic drainage provided effective palliative care and improved quality of life for 44 days despite the poor prognosis. This case highlights the diagnostic value of immunohistochemistry, the potential of early palliative management, and the need to consider neoplastic causes in young cats presenting with pericardial effusion.

## Abstract (plain language summary)

A 1-year-old spayed female American Shorthair cat presented with labored breathing caused by pericardial effusion. Ultrasound revealed a mass between the pericardium and the left ventricular apex. Although temporary improvement followed fluid drainage, the effusion recurred. Further evaluation showed rapid tumor growth, leading to partial pericardiectomy, chest drain placement, and biopsy. Immunohistochemistry excluded mesothelioma and histiocytic sarcoma, confirming an undifferentiated pericardial sarcoma of unknown origin. This rare case, with only one other similar report in a young cat, emphasizes the importance of considering neoplasia in young animals with recurrent pericardial effusion.

## 1. Introduction

Undifferentiated sarcoma is a malignant tumor characterized by the absence of a distinct cellular origin, as determined by morphological and immunohistochemical analyses, and is known for its highly invasive and proliferative behavior [1]. In human medicine, undifferentiated pleomorphic sarcoma (UPS) most commonly arises in soft tissues, while its occurrence in visceral organs, including the heart, is exceedingly rare [2]. In veterinary medicine, reports of undifferentiated sarcomas are limited, and those originating from the pericardium of cats are particularly uncommon [3].

Pericardial effusion (PE) in cats is uncommon relative to other cardiac diseases, but when it occurs, it often signals serious underlying pathology. Necropsy studies indicate a low incidence (approximately 1–2.3%) of significant pericardial effusion in feline hearts [4]. Among live cats, congestive heart failure (CHF) and neoplasia rank among the most common causes of moderate to large effusions. In a study of 83 cats with PE, 45% were due to CHF and 19% due to neoplasia [5]. Similarly, Hall et al. evaluated 164 cats and confirmed CHF and neoplasia as the predominant etiologies [6].

Cardiac tumors in cats are generally rare but are frequently malignant [7,8]. Among these, lymphoma has been identified as the most common type [8]. However, due to the small number of documented cases, detailed information regarding the clinical course, imaging characteristics, and therapeutic management of feline cardiac tumors remains scarce. To date, only a single case of pericardial undifferentiated pleomorphic sarcoma has been reported in a young cat [9].

The diagnosis of undifferentiated sarcoma poses a significant challenge. Cytologic evaluation of pericardial effusion often yields inconclusive results, making histopathological and immunohistochemical examination essential to differentiate this tumor from other neoplastic entities such as mesothelioma, hemangiosarcoma, or lymphoma [10]. Prognosis in cats with pericardial tumors is generally poor, especially for non-lymphomatous neoplasms, owing to rapid disease progression, frequent involvement of myocardium or other thoracic structures, and limited therapeutic options. Many cases are only definitively diagnosed at necropsy [11]. However, early diagnosis and palliative interventions may help alleviate clinical signs and extend quality of life, even when curative treatment is not feasible.

Here, we describe a case of pericardial sarcoma of uncertain origin in a young cat, emphasizing the diagnostic challenges, histopathological and immunohistochemical findings, and the role of thoracic drainage as a palliative therapeutic strategy.

## 2. Case Presentation

### 2.1. Initial Presentation and Clinical Findings

A 1-year-old spayed female American Shorthair cat was presented to the emergency clinic at night with acute onset of labored breathing. Over the previous 30 days, the referring veterinarian had performed thoracocentesis four times to remove presumed pleural effusion. On presentation, the cat weighed 3.16 kg. Mucous membranes were pink with no evidence of cyanosis, but the patient exhibited labored respiration and decreased activity.

**Figure 1:**
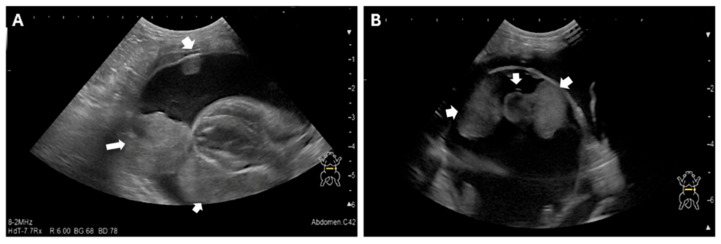
Echocardiographic Images of the Pericardial Mass (**A**) and (**B**) Two-dimensional echocardiography images demonstrate a large volume of anechoic pericardial effusion surrounding the heart. A distinct, heterogeneous soft-tissue mass is visible (within the effusion, adhered to the epicardium), as the presumed cause of the effusion. The internal cardiac chambers and myocardial function were qualitatively assessed as normal. The arrow indicates the intrapericardial mass.

**Figure 2:**
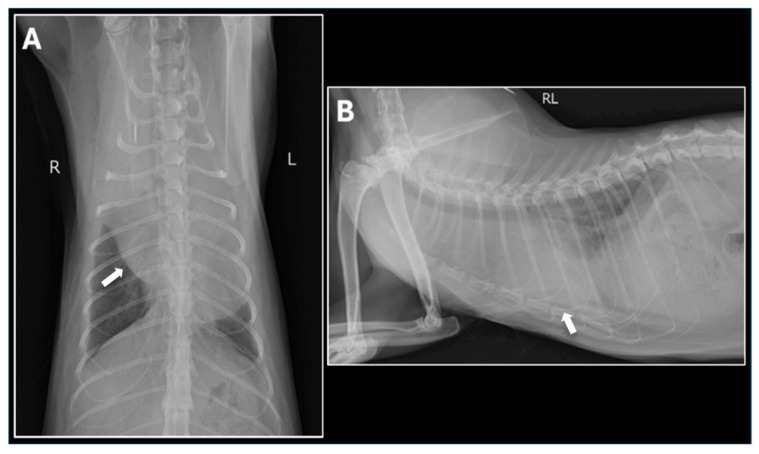
Thoracic Radiographs on Presentation. (**A**) Dorsoventral (DV) view and (**B**) Right Lateral (RL) view of the thorax demonstrate a severely enlarged, rounded cardiac silhouette, characteristic of large-volume pericardial effusion. A mild to moderate volume of pleural effusion is also present, leading to poor visualization of the caudal lung margins. The arrows indicate the presumed location of the heart.

Given the owner’s request for palliative fluid removal, point-of-care ultrasound (POCUS) was performed, revealing significant thoracic fluid accumulation. Thoracocentesis was performed and yielded 225 mL of exudate. Subsequent evaluation determined that the fluid originated from the pericardial sac, confirming severe pericardial effusion rather than pleural effusion. Echocardiography demonstrated normal cardiac function with no evidence of structural cardiac disease typically associated with pericardial effusion (Figure 1, Table 1). However, a mass-like lesion was identified between the pericardium and the left cardiac apex (Figure 2).

Following pericardiocentesis, the cat’s general condition temporarily improved, with better appetite and activity for three days. However, the pericardial effusion rapidly reaccumulated, accompanied by recurrent dyspnea, anorexia, and lethargy.

### 2.2. Referral and Diagnostic Evaluation

**Figure 3:**
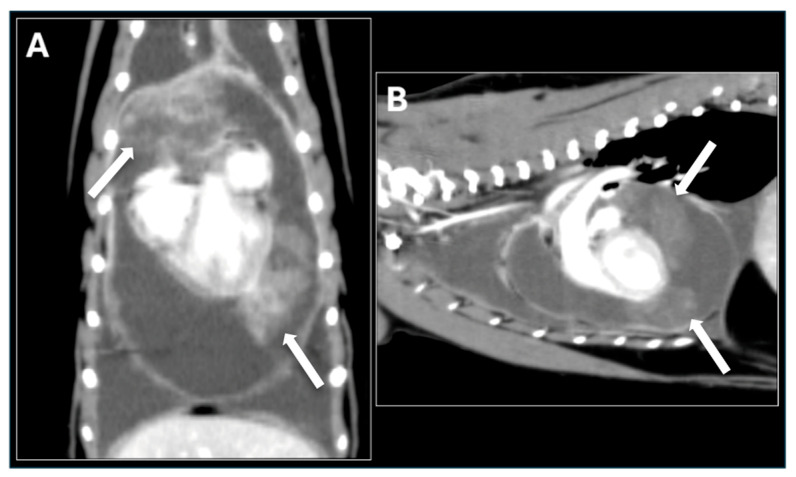
Contrast-enhanced Computed Tomography (CT) Images. (**A**) Dorsal plane and (**B**) Sagittal plane post-contrast CT images of the thorax demonstrate a large, well-defined, heterogeneous mass surrounding the heart. The mass extends from the dorsal aspect of the heart base, along the left cardiac apex, and partially into the cranial heart base. The mass shows moderate, irregular peripheral contrast enhancement and is intimately associated with the cardiac silhouette, consistent with a large pericardial tumor. The arrow indicates the mass.

For further evaluation and treatment, the cat was referred to the Tokyo University of Agriculture and Technology Animal Medical Center. Cytologic examination of the pericardial fluid sediment revealed numerous inflammatory cells but no neoplastic cells. Viral screening tests were negative. Thoracic radiographs showed increased soft tissue opacity in the cranial thorax. Contrast-enhanced computed tomography (CT) demonstrated a well-defined mass extending from the dorsal aspect of the heart base on the left side to the left cardiac apex and cranial heart base (Figure 3). The mass was located within the pericardial sac, accompanied by moderate to severe pericardial effusion. Echocardiography confirmed the presence of the pericardial mass and evidence of right atrial collapse consistent with cardiac tamponade. Fine-needle aspiration (FNA) of the mass was performed but failed to identify definitive neoplastic cells.

### 2.3. Surgical Management and Intraoperative Findings

**Figure 4:**
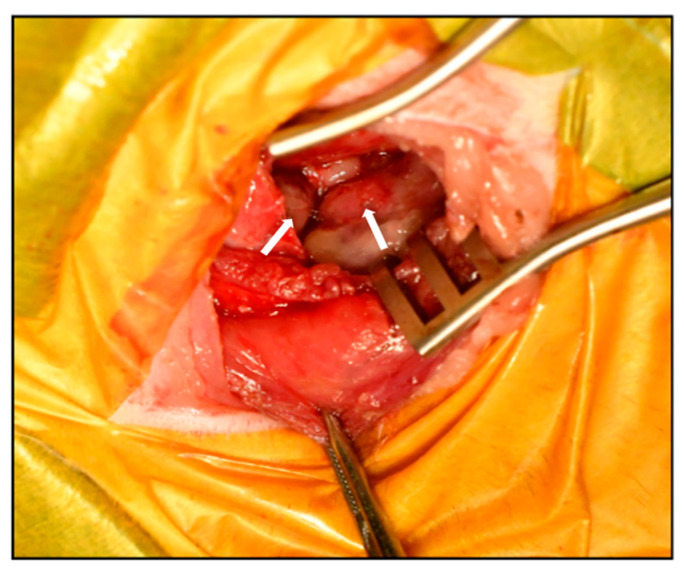
Gross pathological findings during surgery. Intraoperative view demonstrating markedly thickened pericardium with irregular internal surface and multiple small masses visible on the pericardial surface. The arrow indicates the intrapericardial mass.

The therapeutic plan included: (1) placement of a thoracic drain to relieve respiratory distress, (2) definitive diagnosis via tissue biopsy, and (3) partial pericardiectomy to decompress the pericardial space. A left fifth intercostal thoracotomy was performed to partially resect the pericardium and collect biopsy specimens. Intraoperatively, the pleura appeared smooth, and no gross nodules were present on the lung surface. Upon incision of the pericardium, a large volume of fluid was released. The pericardium was markedly thickened with an irregular inner surface, and multiple small masses were observed on both the internal and external pericardial surfaces (Figure 4). Due to abundant neovascularization and tissue fragility, only partial pericardiectomy was feasible. A thoracic drain was placed to facilitate ongoing drainage and potential intrathoracic chemotherapy administration, followed by routine closure of the thoracotomy site.

### 2.4. Histopathology and Immunohistochemistry

**Figure 5:**
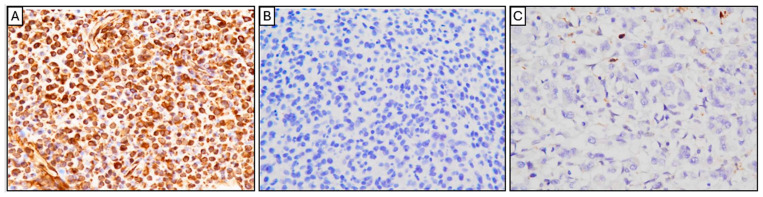
Immunohistochemical (IHC) analysis of the pericardial tumor. The neoplastic cells showed strong, diffuse cytoplasmic and nuclear positivity for vimentin (**A**), confirming mesenchymal origin. The cells were negative for cytokeratin (**B**), ruling out epithelial tumors (such as mesothelioma). The tumor cells were also negative for Iba-1 (**C**), excluding histiocytic sarcoma. (Original magnification: ×400 for all panels).

Multiple dome-shaped to irregular nodules were present on both surfaces of the pericardium, consisting of densely proliferating, highly pleomorphic tumor cells with indistinct cell borders. The cells contained scant to moderate weakly eosinophilic cytoplasm, occasionally with ground-glass-like vacuoles, and round to ovoid nuclei with prominent nucleoli. Mitotic figures were frequent (approximately 30 per 10 high-power fields), including occasional atypical mitoses. Based on the gross findings and clinical course, malignant mesothelioma and histiocytic sarcoma were initially suspected. Histopathological and immunohistochemical (IHC) analyses were subsequently performed. The neoplastic cells were positive for vimentin but negative for cytokeratin and Iba-1 (Figure 5A–C), ruling out both mesothelioma and histiocytic sarcoma. In conjunction with the histopathological morphology, these findings supported a diagnosis of sarcoma of uncertain origin. Although intrathoracic carboplatin chemotherapy was initially considered under the presumption of mesothelioma, treatment was discontinued following the IHC confirmation. Instead, corticosteroid therapy was initiated to stimulate appetite and improve comfort.

### 2.5. Follow-Up and Outcome

**Figure 6:**
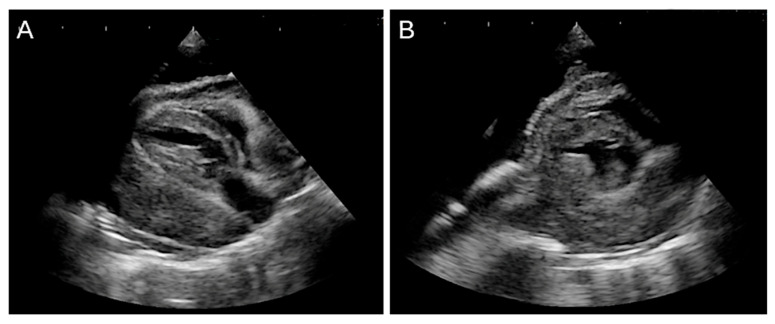
Persistent pleural effusion was observed after surgery. (**A**,**B**) Two-dimensional echocardiographic images show a mass completely occupying the space between the pericardium and the heart, resulting in severe cardiac compression and findings consistent with right atrial collapse.

Post-discharge echocardiography revealed tumor progression, with infiltration from the pericardium into the myocardium (Figure 6). The cat’s appetite fluctuated, and gradual weight loss was observed. Forty-four days after thoracic drain placement, the patient died at home due to respiratory distress.

### 2.6. Necropsy Findings

**Figure 7:**
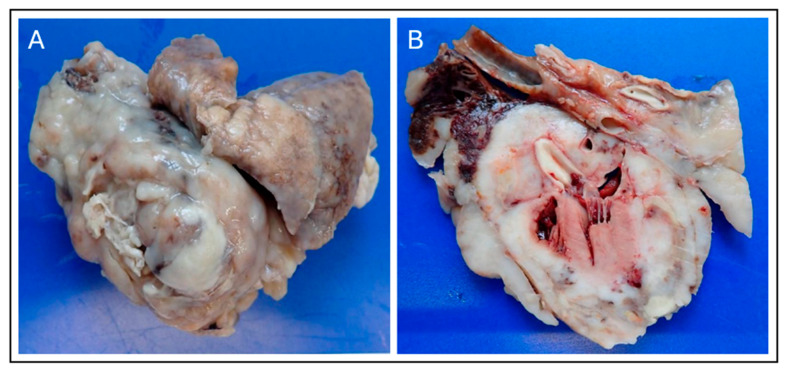
Gross Necropsy Findings of the Pericardial Sarcoma. (**A**) External view of the surgically resected heart and associated mass, demonstrating the large, multilobulated, milky-white to tan appearance of the malignant neoplasm that has replaced and thickened the pericardium. (**B**) Cross-section of the heart and tumor mass. Gross examination confirms extensive tumor infiltration into the epicardium and underlying myocardium, with the milky-white neoplastic tissue diffusely replacing normal cardiac structure, confirming the diagnosis of an advanced primary pericardial sarcoma.

A complete necropsy was performed with the owner’s consent. The pericardium appeared markedly thickened and irregular, with an uneven pleural surface. After fixation, gross examination confirmed extensive tumor infiltration throughout the pericardial and myocardial tissues. Multiple nodular lesions were present in the lungs. The heart was firmly adherent to the tumor, and on sectioning, tumor invasion was evident in the epicardium and myocardium. Macroscopically, the neoplasm consisted of a milky white, solid mass diffusely infiltrating the cranial mediastinum, pericardium, and myocardium, consistent with advanced pericardial sarcoma (Figure 7).

**Figure 8:**
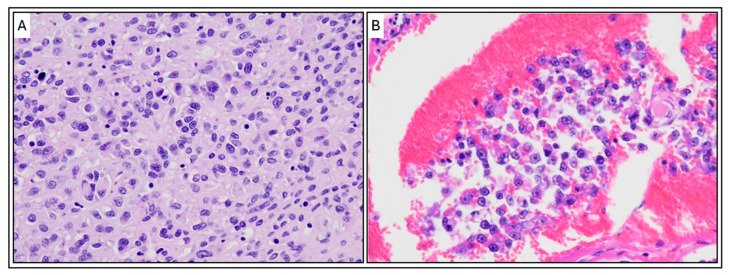
Histopathological Features of the Pericardial Sarcoma (H&E Stain). (**A**) High-magnification micrograph of the tumor tissue. The image shows a highly cellular and proliferative sheet-like growth of malignant, pleomorphic cells. Tumor cells exhibit moderate anisokaryosis (variation in nuclear size) and shape (round, polygonal, or spindle-shaped), with scant to moderate eosinophilic cytoplasm and prominent nucleoli, consistent with a high-grade sarcoma. (**B**) Evidence of vascular invasion. The micrograph demonstrates a cluster of the neoplastic cells invading and filling a blood or lymphatic vessel (intravascular/lymphatic tumor embolus), surrounded by a background of congestion. Magnification: ×400. Scale bar 50 μm.

Histologic examination revealed diffuse and highly proliferative tumor cells thickening the epicardium, with infiltration into the underlying myocardium (Figure 8). In the right ventricle, tumor cells penetrated the entire myocardial wall, reaching the endocardium. The pleura of the lungs showed diffuse thickening due to tumor cell proliferation. Tumor cells infiltrated and congested the entire pulmonary vasculature, including alveolar capillaries, and small tumor nodules were scattered throughout the lung lobes. The alveoli remained aerated, with no evidence of pulmonary edema.

## 3. Discussion

Primary cardiac tumors are extremely rare in both human and veterinary medicine, with sarcomas arising in the pericardium being even more uncommon. In humans, cardiac sarcomas, including undifferentiated pleomorphic sarcoma (UPS), are known for their high invasiveness and are generally associated with poor prognosis [12]. In cats, the majority of reported pericardial tumors are lymphomas or mesotheliomas, while sarcomas in this location are exceedingly rare [13,14]. Due to the scarcity of such reports, it remains challenging to establish standardized diagnostic and treatment strategies.

Previous reports of cardiac tumors in young cats primarily describe intracardiac tumors. Examples include inflammatory myofibroblast tumors diagnosed by immunohistochemistry with a mean age of 2.6 years [15] and a case of B-cell lymphoma in a 3-year-old American Shorthair cat [16]. More recently, a case of epicardial UPS accompanied by pericardial effusion was reported in a young cat [9]. In the present case, the patient was even younger—only one year old—making clinical suspicion of neoplasia more difficult.

Based on the clinical course and imaging findings, malignant mesothelioma was initially suspected. However, immunohistochemical analysis revealed a profile of vimentin positivity, cytokeratin negativity, and Iba-1 negativity, effectively excluding mesothelioma and histiocytic sarcoma. The tumor was therefore diagnosed as an pericardial sarcoma of unknown origin. The clinical course of this case was rapidly progressive. At presentation, the cat exhibited dyspnea and pleural effusion, which was temporarily relieved by thoracocentesis; however, effusion recurred repeatedly. Histopathological examination revealed tumor cells extending from the epicardium to the endocardium in the right ventricle, with widespread infiltration into the pericardium, myocardium, and pulmonary vasculature, indicating a highly malignant neoplasm.

In feline oncology, establishing the primary origin of pericardial sarcomas remains a diagnostic challenge. In the present case, the tumor is thought to have arisen from the epicardium, although the possibility that it originated in the myocardium and subsequently infiltrated the epicardium cannot be ruled out. Accordingly, careful evaluation of the tumor’s primary site is essential. Distinguishing between primary cardiac, pericardial, and metastatic sarcomas often requires a combination of imaging, cytology, histopathology, and a broad immunohistochemical panel [7,17]. In several reports, UPS has demonstrated histological overlap with fibrosarcoma, rhabdomyosarcoma, or leiomyosarcoma, emphasizing the importance of using lineage-specific markers for definitive classification [18]. Desmin or other muscle-specific markers were not included in the immunohistochemical panel, which represents a limitation in definitively excluding rhabdomyosarcoma or other sarcomas with muscular differentiation.

In this case, differentiation between mesothelioma and histiocytic sarcoma was important in selecting treatment, and numerous immunohistochemical staining were required to make a definitive diagnosis of sarcoma. Considering the patient’s prognosis, further immunohistochemical staining was not necessary during his lifetime, so it was not performed, but we believe that additional study is necessary for the future. Furthermore, the tendency for undifferentiated sarcomas to diffusely infiltrate adjacent structures, such as the myocardium and pleura, often complicates surgical resection and contributes to rapid clinical deterioration [8].

Given the cat’s age, this case also raises the question of potential genetic or developmental predisposition to early-onset sarcomas. Although data in veterinary species are scarce, recent studies in human pediatric sarcomas suggest the possible involvement of TP53 and RB1 mutations in undifferentiated soft tissue sarcomas [19]. Future genomic and molecular profiling of feline sarcomas may reveal similar oncogenic mechanisms and provide novel diagnostic or therapeutic targets.

Placement of a thoracic drain enabled extended at-home care and reduced stress from frequent hospital visits, allowing the cat to spend more time with its family. While intrathoracic chemotherapy using agents such as carboplatin or cisplatin has been attempted in cases of mesothelioma in small animals [20], chemotherapy was not pursued in this case due to the immunohistochemical exclusion of mesothelioma and the owner’s preference. Although sarcomas are generally aggressive and associated with a poor prognosis, supportive therapy involving corticosteroids and drain management allowed for 44 days of home-based care. As curative treatment is rarely feasible, early palliative intervention focusing on symptom relief is considered essential in such cases.

From a clinical management perspective, palliative pericardial drainage remains a practical option for maintaining short-term comfort and hemodynamic stability in patients with recurrent effusion. In canine cases, pericardiectomy and drainage have been associated with transient improvement in quality of life but limited impact on overall survival [21,22]. Combining palliative drainage with anti-inflammatory or low-dose chemotherapeutic protocols may help prolong symptom-free intervals, although evidence remains limited.

Diagnosis of pericardial sarcomas relies on advanced imaging combined with early surgical biopsy, as cytology frequently fails to detect neoplastic cells. Although complete surgical excision is considered the most effective therapeutic option, it is rarely achievable due to extensive infiltration of the pericardium and myocardium. In theory, adjunctive treatments such as radiotherapy or systemic chemotherapy (e.g., anthracycline-based protocols or tyrosine kinase inhibitors such as toceranib) may provide benefit, but evidence in feline cardiac sarcomas is lacking, and their efficacy in young patients remains undetermined.

This case contributes several novel insights to the veterinary literature.

First, pericardial sarcomas in cats are exceedingly rare [9], and the extremely young age of the patient underscores that neoplastic disease should remain in the differential diagnosis for pericardial effusion even in juvenile cats.

Second, this case provides a detailed account of clinical findings, diagnostic imaging, histopathology, and immunohistochemistry in feline pericardial sarcoma.

Third, continuous documentation from the initial clinical presentation through necropsy offers a comprehensive view of disease progression.

Finally, the use of a thoracic drain enabled long-term palliative care at home, improving the patient’s quality of life and presenting a noteworthy palliative strategy for managing feline cardiac sarcomas.

## 4. Conclusions

This case describes a rare pericardial sarcoma of uncertain origin in a one-year-old cat. Definitive diagnosis was achieved through surgical biopsy and immunohistochemical analysis, as cytology of the pericardial effusion failed to detect tumor cells. Histopathology revealed extensive infiltration of the pericardium, myocardium, and pulmonary vasculature, indicating an aggressive nature. Thoracic drain placement provided both diagnostic and palliative benefits by alleviating respiratory distress and reducing hospital stress. Even in young cats, neoplasia should be considered in cases of recurrent pericardial effusion, and early tissue biopsy is recommended due to the potential absence of tumor cells in the fluid. Accumulation of similar cases is essential to enhance understanding, diagnosis, and management. Future studies are needed to evaluate the potential roles of radiotherapy and systemic chemotherapeutic agents, including anthracyclines and tyrosine kinase inhibitors, in managing feline pericardial sarcomas.

## Figures and Tables

**Table 1:** Results of echocardiographic examination.

Parameters	Measurement
IVSd (mm)	4.8
LVIDd (mm)	9.0
LVPWd (mm)	7.1
IVSs (mm)	5.9
LVIDs (mm)	4.0
LVPWs (mm)	8.8
FS (%)	56.1
RVOTv (cm/s)	58.0
LVOTv (cm/s)	65.5
Ev (cm/s)	91.0
E/A	1.80
s’sep (cm/s)	5.3
e’sep (cm/s)	6.5
E/e’sep	13.92
s’lat (cm/s)	4.8
e’lat (cm/s)	4.5
E/e’ lat	20.25

IVSd: interventricular septum thickness at end-diastole; LVIDd: left ventricular end-diastolic dimension; LVPWd: left ventricular posterior wall thickness at end-diastole; IVSs: interventricular septum thickness at end-systole; LVIDs: left ventricular end-systole dimension; LVPWs: left ventricular posterior wall thickness at end-systole; FS: fractional shortening; RVOTv: right ventricular outflow tract velocity; LVOTv: left ventricular outflow tract velocity; Ev: E wave velocity; E/A: E wave to A wave ratio; s’sep: systolic peak velocity of the septal mitral annulus; e’sep: early diastolic velocity of the septal mitral annulus; s’lat: systolic peak velocity of the lateral mitral annulus; e’sep: early diastolic velocity of the lateral mitral annulus.

## Data Availability

The original contributions presented in the study are included in the article; further inquiries can be directed to the corresponding author.
